# Crystal structure and magnetism in the *S* = 1/2 spin dimer compound NaCu_2_VP_2_O_10_

**DOI:** 10.1107/S2052252520005655

**Published:** 2020-05-27

**Authors:** Daisuke Urushihara, Sota Kawaguchi, Koichiro Fukuda, Toru Asaka

**Affiliations:** a Division of Advanced Ceramics, Nagoya Institute of Technology, Nagoya 466-8555, Japan; b Frontier Research Institute for Materials Science, Nagoya Institute of Technology, Nagoya 466-8555, Japan

**Keywords:** single-crystal X-ray diffraction, electron diffraction, spin dimer compounds, magnetic susceptibility

## Abstract

The crystal structure of the spin dimer magnet NaCu_2_VP_2_O_10_ was determined using single-crystal X-ray diffraction and electron diffraction. NaCu_2_VP_2_O_10_ displayed a non-centrosymmetric orthorhombic *C*222_1_ structure with *a* = 6.13860 (10) Å, *b* = 14.4846 (3) Å and *c* = 8.2392 (2) Å. The layered structure comprised CuO_4_ plaquettes, VO_6_ octahedra and PO_4_ tetrahedra. A pair of CuO_4_ plaquettes formed Cu_2_O_6_ structural dimers through edge sharing. The Cu–Cu network formed a distorted puckered-layer structure with pseudo-one-dimensional characteristics. Maximum magnetic susceptibility was observed at ∼60 K and NaCu_2_VP_2_O_10_ became non-magnetic upon further cooling. The spin gap between the spin-singlet non-magnetic ground state and triplet excited state was estimated to be 43.4 K. Thus, NaCu_2_VP_2_O_10_ was assumed to be an alternating chain system with a singlet ground state of dimer origin. The V^5+^ ions in the VO_6_ octahedra showed large off-centre displacements along the [110] direction in the primitive perovskite structure, which were attributed to the pseudo-Jahn–Teller distortion of *d* ^0^ transition metals.

## Abstract 2

The spin dimer magnet NaCu_2_VP_2_O_10_ was discovered, and its crystal structure was determined using single-crystal X-ray diffraction and electron diffraction. Maximum magnetic susceptibility was observed at ∼60 K and NaCu_2_VP_2_O_10_ became non-magnetic upon further cooling.

## 1. Introduction   

Quantum-spin systems have attracted considerable attention since the discovery of characteristic quantum phenomena such as superconductivity and quantum-spin liquids (Lee, 2008 ▸; Balents, 2010 ▸). Spin dimer systems are representative materials that exhibit quantum-spin fluctuations. In such systems, magnetic ions are often coupled with antiferromagnetic exchange interactions, resulting in the formation of spin dimers. Dimers are coupled with neighbouring dimers via weak exchange interactions. With increasing interactions between dimers in low-dimensional systems, spin dimer compounds can be considered as an alternating chain system. Dimerized quantum magnets usually display an energy gap between spin-singlet non-magnetic ground and triplet excited states; furthermore, the inter- and intra-dimer interactions caused by the arrangement of magnetic ions affect the energy gap.

In the spin dimer system of Cu^2+^ (*S* = 1/2), dimerized Cu^2+^ often shows characteristic atomic arrangements; Cu^2+^, which possesses a 3*d* ^9^ configuration, exhibits Jahn–Teller distortion owing to the occupied *d*_3*z*2–*r*2_ orbital and sometimes forms a CuO_4_ plaquette or distorted CuO_6_ octahedron. A CuO_4_ plaquette is two-dimensional because its ligands prefer planar coordination. Thus, Cu_2_O_6_ dimers formed by edge-sharing CuO_4_ plaquettes show various arrangements which are related to interactions between Cu ions. These Cu_2_O_6_ dimers have been identified in compounds such as SrCu_2_(BO_3_)_2_ (Smith & Keszler, 1991 ▸; Kageyama *et al.*, 1999 ▸), Cu_2_P_2_O_7_ (Effenberger, 1990 ▸; Janson *et al.*, 2011 ▸) and BaCu_2_V_2_O_8_ (Vogt & Müller-Buschbaum, 1990 ▸; Klyushina *et al.*, 2016 ▸). SrCu_2_(BO_3_)_2_ represents a typical two-dimensional orthogonal dimer system. In SrCu_2_(BO_3_)_2_, Cu_2_O_6_ dimers are located orthogonally along the [110] direction in a tetragonal system and connected through BO_3_ triangles. In Cu_2_P_2_O_7_, Cu_2_O_6_ dimers are located parallel to the *b* axis in the monoclinic system. The Cu–Cu network in Cu_2_P_2_O_7_ has a distorted two-dimensional honeycomb structure. In contrast, BaCu_2_V_2_O_8_ has three-dimensional arrangements of Cu_2_O_6_ dimers and its Cu–Cu network adopts pseudo-one-dimensional screw chains along the *c* axis. Both arrangements of Cu_2_O_6_ dimers and Cu–Cu networks strongly affect quantum states. Thus, it is important to discover other examples of spin dimer systems with a finite spin gap to an excited state.

In this study, we synthesize the spin dimer compound NaCu_2_VP_2_O_10_, the crystal structure of which was determined using single-crystal X-ray diffraction (XRD) and electron diffraction. The obtained crystal structure is layered, and it comprises corner-sharing Cu_2_O_6_ dimers, VO_6_ octahedra and PO_4_ tetrahedra. Magnetic susceptibility measurements of NaCu_2_VP_2_O_10_ reveal that it exhibits a non-magnetic ground state and a spin gap between the ground and excited states. We clarify that NaCu_2_VP_2_O_10_ is an alternating chain system and discuss the relationship between its crystal structure and magnetic properties.

## 2. Experimental section   

### 2.1. Synthesis   

Polycrystalline samples were synthesized by a solid-state reaction. A mixture of equal amounts of NaVO_3_ and Cu_2_P_2_O_7_ was sintered at 823 K for 20 h and then 923 K for 10 h. To grow single crystals, the sintered sample was heated at 1023 K for 2 h and then cooled to 923 K at a rate of 1 K/ h. The obtained crystals had a columnar shape with a diameter of ∼50 µm.

### 2.2. Electron-probe microanalysis   

The chemical composition of the polycrystalline materials was measured using an electron-probe microanalyzer (JXA-8230, JEOL). The prepared samples were polished to form flat surfaces. The normalized Na:Cu:V:P ratio for the three grains was determined to be 0.87 (12):2.014 (7):1.094 (3):2.022 (9), which is close to the stoichiometric chemical composition (Na:Cu:V:P ratio of 1:2:1:2).

### 2.3. Powder X-ray diffraction   

To determine the phase purity and estimate the basic structure of the synthesized compounds, we collected powder XRD patterns using a powder X-ray diffractometer (X’Pert Pro Alpha-1, Panalytial) equipped with a high-speed detector and Cu *K*α_1_ X-ray source (45 kV, 40 mA). The scanning range of diffraction angles (2θ) was 5–145°, which was adequate for indexing the diffraction peaks. We confirmed that the samples did not contain any impurity phases by comparing the measured powder XRD patterns with a simulated pattern of the refined structure model of NaCu_2_VP_2_O_10_ (see Fig. S1 in the Supporting information).

### 2.4. Electron diffraction   

Selected-area electron diffraction (SAED) measurements were performed using a transmission electron microscope (JEM-ARM200F, JEOL) operated at 200 kV. The specimen was prepared by crushing the polycrystals; the particles were deposited on a copper grid with a holey carbon support film. We determined the space group by testing the extinction rules of the sample using SAED.

### 2.5. Single-crystal X-ray diffraction   

Diffraction data were collected using a single-crystal X-ray diffractometer (D8 VENTURE, Bruker) equipped with a complementary metal oxide semiconductor detector and Mo *K*α X-ray source (50 kV, 1 mA). A single crystal with a diameter of ∼50 µm was mounted on a borosilicate glass needle using an adhesive. Lattice constants were determined using the *SAINT* program (Bruker, 2015 ▸) and multi-scan absorption correction was carried out using the *SADABS* program (Bruker, 2015 ▸). The initial structure model was calculated using the *SUPERFLIP* program based on the charge-flipping algorithm (Palatinus & Chapuis, 2007 ▸). Crystal structure analysis was carried out using the *JANA2006* program package (Petricek *et al.*, 2014 ▸) and the analysed crystal structure was visualized using the *VESTA* program (Momma & Izumi, 2011 ▸).

### 2.6. Magnetic susceptibility   

The magnetic susceptibility of the polycrystalline materials was measured using a superconducting quantum-interference device magnetometer (MPMS, Quantum Design). Magnetization was obtained at 2–400 K in an applied field of 1 T.

### 2.7. Thermal analysis   

The heat capacity of the polycrystalline materials was measured using a physical property measurement system (PPMS, Quantum Design). The temperature dependence of the heat capacity was measured at 2–300 K using a thermal-relaxation method. No thermal anomalies were observed in the investigated temperature range.

## 3. Results   

### 3.1. Space group determination   

**Figure 1:**
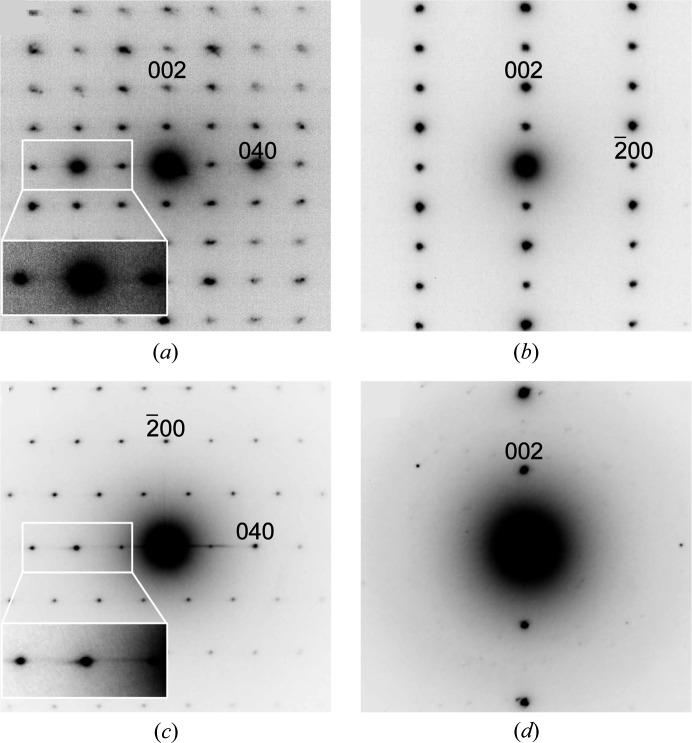
(*a*) [100] zone axis, (*b*) [010] zone axis and (*c*) [001] zone axis SAED patterns of NaCu_2_VP_2_O_10_. (*d*) 00*l* systematic reflections of NaCu_2_VP_2_O_10_.

We measured the powder XRD pattern of NaCu_2_VP_2_O_10_, which could be indexed to an orthorhombic unit cell. According to the indices obtained from the powder XRD patterns, the zone axes of the incident electron beams were identified in the SAED patterns. Fig. 1 ▸ shows the SAED patterns collected under several electron beams with different incidences. In the [100] and [010] zone axis SAED patterns [Figs. 1 ▸(*a*) and 1 ▸(*b*)], only diffraction spots with indices of *k* = 2*n* and *h* = 2*n* were observed. The [001] zone axis SAED pattern [Fig. 1 ▸(*c*)] shows an extinction rule of *h* + *k* = 2*n*, which contains both *k* = 2*n* and *h* = 2*n* in the [100] and [010] zone axis SAED patterns. Such extinction rules indicate the *C* lattice symmetry (*hkl*: *h* + *k* = 2*n*) of the orthorhombic system. We then observed the diffraction spots of the 00*l* condition by tilting the specimen to remove the multiple reflections at forbidden reflection positions, which may appear in a crystal structure with screws or grid planes [Fig. 1 ▸(*d*)]. The reflections in the 00*l* condition were indexed as *l* = 2*n*, which represents a twofold screw parallel to the *c* axis (00*l*: *l* = 2*n*). The extinction rule was identical to that determined from the single-crystal XRD data, which was based on the kinematical theory of diffraction [Figs. S2(*a*)–S2(*c*)]. Analysis of the SAED patterns revealed that the space group of NaCu_2_VP_2_O_10_ was *C*222_1_ and non-centrosymmetric.

### 3.2. Crystal structure determination from XRD data   

**Figure 2:**
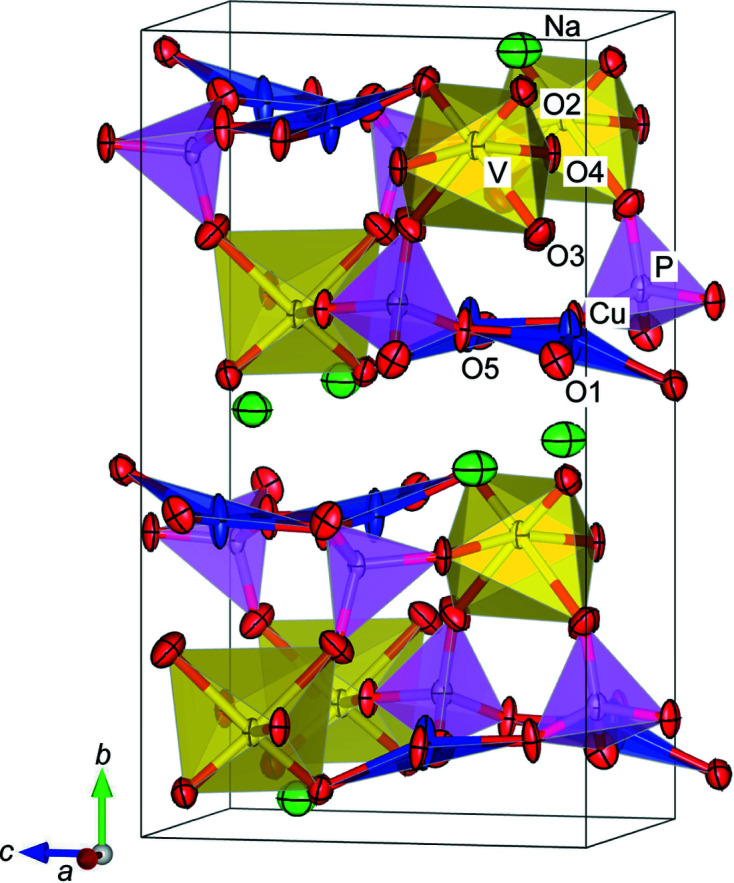
Crystal structure model of NaCu_2_VP_2_O_10_. Atom colours: Na (green), Cu (blue), V (yellow), P (pink) and O (red). Ellipsoids are set at a 90% probability level.

Single-crystal XRD data obtained for NaCu_2_VP_2_O_10_ were indexed to an orthorhombic cell, consistent with the SAED patterns. The determined unit-cell dimensions of NaCu_2_VP_2_O_10_ were *a =* 6.13860 (10) Å, *b* = 14.4846 (3) Å and *c* = 8.2392 (2) Å. The initial structure model was determined using the charge-flipping method. This model represents nine independent sites in the unit cell. The Na site was at the Wyckoff position 4*b* (Na), the Cu site was at 8*c* (Cu), the V site was at 4*b* (V), the P site was at 8*c* (P) and the O sites were at 8*c* (O1–O5). We successfully refined all site-coordination and anisotropic atomic displacement parameters (*U*). The reliability indices were *R* = 2.33% and *wR* = 7.62%. The *U* values were adequate at all sites. The refined crystal structure model is shown in Fig. 2 ▸; the crystal data, structural parameters and atomic distances are summarized in Tables 1 ▸, 2 ▸ and 3 ▸, respectively.

The average bond distances of 〈Na–O〉, 〈Cu–O〉, 〈V–O〉 and 〈P–O〉 in the crystal structure of NaCu_2_VP_2_O_10_ were 2.5883, 1.9713, 1.9230 and 1.5343 Å, respectively, which are in good agreement with the bond distances estimated by combinations of the effective ionic radii of the respective ions of 2.58, 1.97, 1.94 and 1.57 Å (Shannon, 1976 ▸). The valence of each site was estimated using the bond valence sum (BVS) method, which is used to calculate valence from experimental parameters and bond distances (Brese & O’Keeffe, 1991 ▸). The calculated BVS values of the Na, Cu, V and P sites were 1.07, 1.84, 5.08 and 4.84, respectively. The average bond distances and BVS values were appropriate, which suggests that the structure analysis was successfully performed. As shown in Fig. 2 ▸, Cu ions display characteristic highly anisotropic ellipsoids along the *b* direction. In general, Cu ions in CuO_4_ plaquettes prefer the anisotropic displacement perpendicular to the CuO_4_ plane because there are no apical oxygen ions (Effenberger, 1990 ▸; Smith & Keszler, 1991 ▸). Furthermore, NaCu_2_VP_2_O_10_ has enough space for Cu ions to fluctuate along the *b* direction. The anisotropic ellipsoids of the Cu ions can be interpreted from crystallographic considerations. The Flack parameter, which is an index of the ratio of inversion structure, was determined to be 0.018 (14) (Flack & Bernardinelli, 1999 ▸). Therefore, the measured single crystal is regarded as a monodomain crystal with respect to the inversion twin.

### 3.3. Magnetic susceptibility   

**Figure 3:**
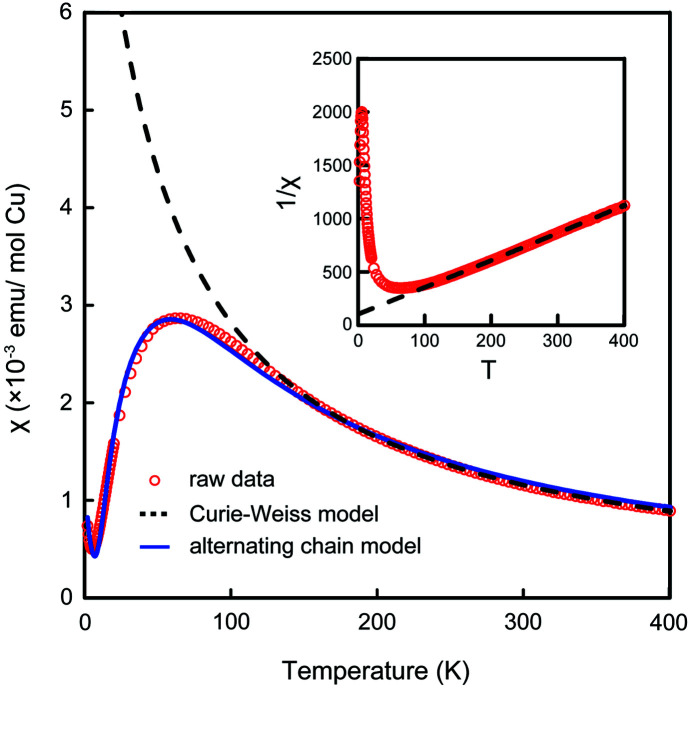
Temperature dependence of the magnetic susceptibility χ of NaCu_2_VP_2_O_10_. Red open symbols represent raw data. The blue solid and black dashed lines show the fitting curves of the alternating chain and Curie–Weiss models. The inset shows the temperature dependence of 1/*χ*.

Fig. 3 ▸ shows the temperature dependence of the magnetic susceptibility measured at 1 T using a single-phase polycrystalline NaCu_2_VP_2_O_10_ sample. The maximum magnetic susceptibility was observed at ∼60 K and the magnetic susceptibility became close to zero upon further cooling. The Curie–Weiss fitting using χ(*T*) = *C*/(*T − θ*) + χ_0_ for the high-temperature region (>200 K) represents the Weiss temperature of θ = −41.9 K and Curie constant of *C* = 4.04 × 10^−1^ emu K/ mol Cu. The inset in Fig. 3 ▸ shows the temperature dependence of 1/χ, which represents a straight line owing to Curie paramagnetism in the high-temperature region. Both the effective magnetic moment of μ_eff_ = 1.80μ_B_ and the *g* factor of 2.07 are decent values for Cu^2+^ (*S* = 1/2) compounds. Fitting for the magnetic susceptibility using the isolated spin dimer model (Bleaney & Bowers, 1952 ▸) resulted in failure (Fig. S3). Alternatively, the data can be fitted in the full range using an alternating Heisenberg chain model (Johnston *et al.*, 2000 ▸) using the expression χ(*T*) = *N*_A_ *g* ^2^μ_B_^2^/*k*_B_ *J* × χ^*^(*α, T*) + *C*_imp_/(*T − θ*_imp_) + χ_0_. Here, *N*_A_, μ_B_ and *k*_B_ are Avogadro number, Bohr magneton and Boltzmann constant, respectively. The exchange parameter *J*, alternation parameter α ( = *J*′/*J*), *C*_imp_, θ_imp_, and χ_0_ are the fitting parameters. When α = 0 and α = 1, the function represents the isolated spin dimer and the uniform chain models, respectively. A small impurity Curie–Weiss contribution, which appeared because of magnetic impurities or defects of Cu^2+^ in NaCu_2_VP_2_O_10_, was observed below ∼5 K. We obtained a Weiss temperature of θ_imp_ = −2.38 K and Curie constant of *C*_imp_ = 3.72 × 10^−3^ emu K/ mol Cu, corresponding to 0.99% of nearly free *S* = 1/2 impurities; χ_0_ represents the temperature-independent term of −2.35 × 10^−5^ emu/ mol Cu. We obtained *J* = 99.3 K, α = 0.72 and a *g* factor of 2.12 in the *S* = 1/2 alternating chain model. This α value indicates that there are non-negligible interactions between the dimers. Using the relationship Δ ≃ *J*(1 − α)^3/4^(1 + α)^1/4^, we estimated that the spin gap Δ was 43.4 K. In the thermal analysis, the heat capacity indicated that there were no anomalies below 100 K (Fig. S4). This result suggests that the long-range magnetic order did not evolve and that changes in the magnetic susceptibility were not related to a conventional phase transition. The crystal structure and magnetic susceptibility of NaCu_2_VP_2_O_10_ indicated that it is a spin-gap system.

## 4. Discussion   

**Figure 4:**
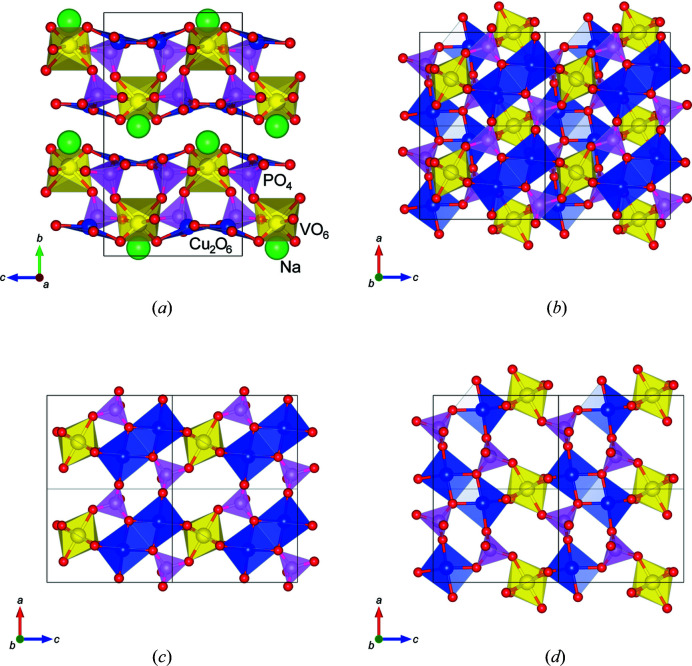
Crystal structure models of (*a*) NaCu_2_VP_2_O_10_ viewed from the *a* axis, (*b*) the local structure of a single layered structure, (*c*) the upper layer unit and (*d*) the lower layer unit.

Fig. 4 ▸(*a*) shows the crystal structure model of NaCu_2_VP_2_O_10_. The layered structure consisted of Cu_2_O_6_ dimers, VO_6_ octahedra and PO_4_ tetrahedra, which were connected through corner sharing. The Na ions were located between the polyhedral layers. As shown in Figs. 1 ▸(*a*) and 1 ▸(*c*), the weak diffuse streak scattering along the [010] direction indicates the presence of stacking faults in the layered structure. The Cu_2_O_6_ dimers were almost parallel to the *ac* plane in each layer. Fig. 4 ▸(*b*) depicts the partial structure of one polyhedral layer viewed from the [010] direction. These layers were composed of two-layer units [Figs. 4 ▸(*c*) and 4 ▸(*d*)]. The two-layer units were related to the twofold screw parallel to the *c* axis, which was constrained by the symmetry of the crystal structure. Each layer unit was connected by corner-sharing VO_6_ octahedra and PO_4_ tetrahedra. Fig. 4 ▸(*c*) displays one of the polyhedral layer units, in which the Cu_2_O_6_ dimers were connected to two VO_6_ octahedra and four PO_4_ tetrahedra. The Cu_2_O_6_ dimers lay in a line approximately parallel to the [101] direction. The PO_4_–VO_6_–PO_4_ polyhedral clusters alternated with Cu_2_O_6_ dimers to fill the space. In the other polyhedral layer unit, Cu_2_O_6_ dimers lay in a line almost along the [10 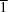] direction [Fig. 4 ▸(*d*)].

**Figure 5:**
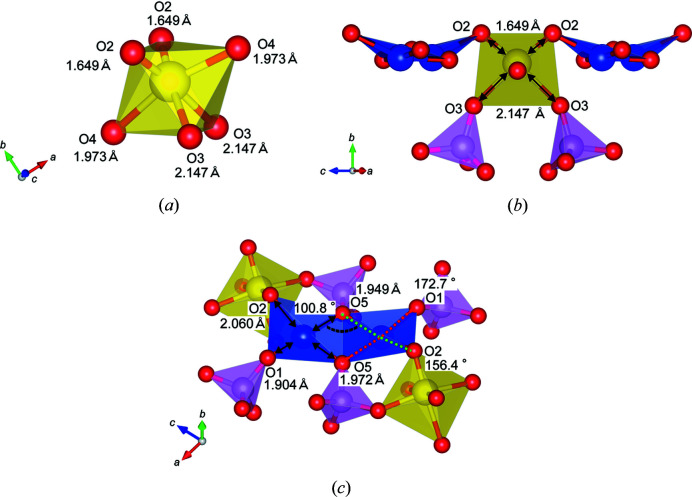
Local structure models of (*a*) a VO_6_ octahedron, (*b*) a corner-sharing octahedron and (*c*) a Cu_2_O_6_ dimer of NaCu_2_VP_2_O_10_.

Fig. 5 ▸(*a*) displays the VO_6_ octahedron in NaCu_2_VP_2_O_10_. The VO_6_ octahedron showed anisotropic V–O bond distances. In particular, the V–O2 bond distance of 1.649 Å was shorter than that of the other bonds in the VO_6_ octahedron. Furthermore, the V–O2 bond distance was not in good agreement with the effective ionic radius of V^5+^ in sixfold coordination {*r*[V^5+^(6)] + *r*[O^2−^(6)]} of 1.94 Å (Shannon, 1976 ▸); this bond distance suggests that hybridization occurred between V^5+^ and O^2−^. Fig. 5 ▸(*b*) also shows anisotropic bond distances, which indicate the large off-centre displacement of V ions. The pseudo-Jahn–Teller effect occurs in the *d* ^0^ transition-metal octahedra when the empty *d* orbitals of the metal form hybrid orbitals with the filled *p* orbitals of the ligands (Bersuker, 2013 ▸; Kunz & Brown, 1995 ▸; Halasyamani & Poeppelmeier, 1998 ▸; Urushihara *et al.*, 2019 ▸). Therefore, V^5+^ ions with a *d* ^0^ configuration can display the pseudo-Jahn–Teller effect. It has been reported that in some vanadium oxides, V^5+^ ions in VO_6_ octahedra show off-centre displacements in the [110] or [100] direction in simple cubic perovskites (Zavalij & Whittingham, 1999 ▸; Halasyamani, 2004 ▸). In NaCu_2_VP_2_O_10_, V^5+^ ions in the VO_6_ octahedra also exhibit an off-centre displacement in the [110] direction in the simple cubic perovskite notation. Here, we defined that the direction of the V—O4 bonds is the [001] direction in the simple cubic perovskite. V^5+^ moves toward the edge of O2 ions connected to the Cu_2_O_6_ dimer, as shown in Fig. 5 ▸(*b*). The off-centre displacement would arise from the Coulomb repulsion between the higher valence V^5+^ and P^5+^ ions and pseudo-Jahn–Teller distortion. In potassium vanadium selenite K(VO_2_)_3_(SeO_3_)_2_, V^5+^ ions also represent an off-centre displacement along the [110] direction in the simple cubic perovskite notation (Harrison *et al.*, 1995 ▸). Two of the six V–O bond distances in the VO_6_ octahedron are much shorter than those expected based on the effective ionic radius. These shorter V—O bonds could be owing to the off-centre displacement towards the octahedral edge, which is accompanied by hybridization between V^5+^ and O^2−^, as with NaCu_2_VP_2_O_10_ compounds.

Fig. 5 ▸(*c*) shows the local structure around the Cu_2_O_6_ dimer. The Cu–O2 bond distance was slightly longer than the other Cu–O bond distances. The green and red dashed lines represent ∠O5—Cu—O2 and ∠O5—Cu—O1 bond angles, which were determined to be 156.4° and 172.7°, respectively. The O2 ions are offset from the prescribed position of the planar CuO_4_ plaquette. This also suggests that O2 ions strongly connect with V^5+^, and the bonding state is different from that of other O ions connected to P^5+^. Thus, NaCu_2_VP_2_O_10_ is expected to exhibit complicated interactions between Cu ions because of Cu—O—V—O—Cu superexchange interactions.

**Figure 6:**
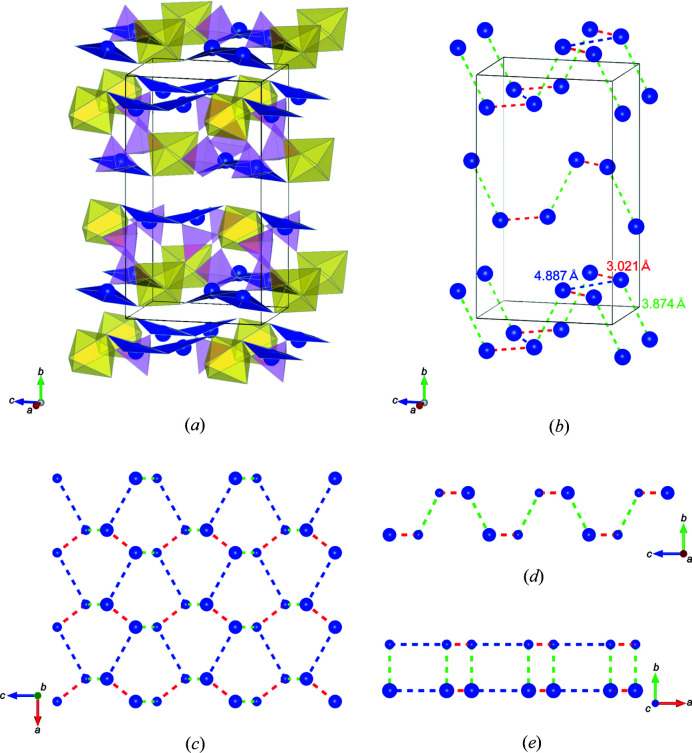
(*a*) Crystal structure model of NaCu_2_VP_2_O_10_. Na ions are omitted. (*b*) Cu–Cu network and local structures of a single layer of the Cu–Cu network along the (*c*) [010], (*d*) [100] and (*e*) [001] directions. Large and small Cu ions represent the atomic positions at the front and back, respectively. The red, green and blue dashed lines indicate the first-, second- and third-nearest-neighbour Cu–Cu bond distances, respectively.

Fig. 6 ▸ shows the Cu–Cu networks, which are arrangements of magnetic ions. The Cu ions form a puckered-layer structure, which has also been observed in other two-dimensional materials such as black phospho­rus (Brown & Rundqvist, 1965 ▸; Liu *et al.*, 2014 ▸). As shown in Figs. 6 ▸(*a*) and 6 ▸(*b*), the first-nearest-neighbour connection is the Cu pairs in Cu_2_O_6_ dimers, which have a distance of 3.021 Å. The dimer bridging angle ∠Cu—O5—Cu is 100.8° [Fig. 5 ▸(*c*)]; therefore, it is reasonable to suppose that the intradimer exchange interaction is antiferromagnetic (Crawford *et al.*, 1976 ▸). The second-nearest-neighbour Cu–Cu connection distance was 3.874 Å and the third-nearest-neighbour connection distance was 4.887 Å. Figs. 6 ▸(*c*)–6 ▸(*e*) display the single layer Cu–Cu network viewed from different directions. The armchair and zigzag directions are along the *c* and *a* axes, respectively. For a prototypical puckered-layer structure, such as that of black phospho­rus, two connections in the layer have the same distances. In contrast, the connections in NaCu_2_VP_2_O_10_ have different distances, which correspond to the first- and third-nearest-neighbour connections. Therefore, the Cu–Cu network displays a highly distorted puckered-layer structure. As a result, the Cu–Cu network can be regarded as a pseudo-one-dimensional system; that is, the Cu–Cu chains along the armchair direction correlate with each other. Thus, NaCu_2_VP_2_O_10_ represents a new type of spin dimer compound with a pseudo-one-dimensional system.

## 5. Conclusions   

In this study, we synthesized the spin dimer compound NaCu_2_VP_2_O_10_. Using selected-area electron diffraction, the space group of NaCu_2_VP_2_O_10_ was revealed to be *C*222_1_. The crystal structure of NaCu_2_VP_2_O_10_ consisted of a layered structure containing Cu_2_O_6_ dimers, VO_6_ octahedra and PO_4_ tetrahedra. Furthermore, temperature-dependent magnetic susceptibility measurements revealed that NaCu_2_VP_2_O_10_ has a non-magnetic ground state and spin gap. V^5+^ in the VO_6_ octahedra exhibited off-centre distortion caused by the pseudo-Jahn–Teller effect. The hybridization between V and O2 led to complicated interactions via the Cu—O—V—O—Cu path. The crystal structure and magnetic susceptibility results suggest that NaCu_2_VP_2_O_10_ is a new quantum-spin system owing to dimerized Cu^2+^.

## Supplementary Material

Crystal structure: contains datablock(s) global, I. DOI: 10.1107/S2052252520005655/fc5043sup1.cif

Structure factors: contains datablock(s) I. DOI: 10.1107/S2052252520005655/fc5043sup2.hkl

Supporting information. DOI: 10.1107/S2052252520005655/fc5043sup3.pdf

CCDC reference: 1976933

## Figures and Tables

**Table 1:** Crystal data and XRD conditions for NaCu_2_VP_2_O_10_

Chemical formula	NaCu_2_VP_2_O_10_
Space group	*C*222_1_ (No. 20)
*a* (Å)	6.13860 (10)
*b* (Å)	14.4846 (3)
*c* (Å)	8.2392 (2)
*V* (Å)^3^	733.58 (3)
*Z*	4
*Dx* (Mg m^−3^)	3.84
	
2θ range	<60.94
Observed reflection	13034
Unique reflection	1110
*R*_int_	0.0254
Collection range	−8 ≤ *h* ≤ 8
	−20 ≤ *k* ≤ 20
	−11 ≤ *l* ≤ 11
*R* (*F* ^2^ > 3σ)	0.0233
*wR* (*F* ^2^)	0.0762

**Table 2:** Structural parameters and atomic displacement parameters of NaCu_2_VP_2_O_10_

Site	Wyckoff position	*g*	*x*	*y*	*z*	*U*_eq_ (Å^2^)
Na	4*b*	1	0	0.46651 (11)	1/4	0.0198 (5)
Cu	8*c*	1	0.15209 (6)	0.11957(3)	0.10586 (4)	0.01583 (12)
V	4*b*	1	0	0.86680 (4)	1/4	0.0072 (2)
P	8*c*	1	0.34633 (11)	0.16200 (4)	0.45456 (7)	0.0066 (2)
O1	8*c*	1	0.0438 (4)	0.39928 (14)	0.5749 (2)	0.0119 (5)
O2	8*c*	1	0.1072 (3)	0.06005 (13)	0.8811 (2)	0.0107 (5)
O3	8*c*	1	0.1107 (4)	0.2370 (2)	0.9171 (2)	0.0153 (6)
O4	8*c*	1	0.2228 (3)	0.34868 (13)	0.1330 (2)	0.0104 (5)
O5	8*c*	1	0.3418 (3)	0.37507 (14)	0.8450 (2)	0.0117 (5)
						
Site	*U*_11_	*U*_22_	*U*_33_	*U*_12_	*U*_13_	*U*_23_
Na	0.0207 (9)	0.0159 (7)	0.0229 (9)	0	0.0051 (8)	0
Cu	0.0051 (2)	0.0363 (2)	0.0061 (2)	0.00172 (14)	0.00042 (13)	0.00126 (13)
V	0.0071 (3)	0.0092 (3)	0.0051 (3)	0	−0.0001 (2)	0
P	0.0046 (3)	0.0099 (3)	0.0054 (3)	−0.0002 (2)	0.0007 (2)	0.0002 (2)
O1	0.0042 (9)	0.0168 (8)	0.0146 (10)	−0.0010 (6)	−0.0016 (7)	−0.0029 (7)
O2	0.0124 (9)	0.0109 (8)	0.0088 (8)	−0.0002 (6)	−0.0005 (7)	0.0009 (7)
O3	0.0162 (10)	0.0167 (9)	0.0131 (10)	−0.0043 (7)	0.0024 (8)	−0.0056 (7)
O4	0.0076 (9)	0.0191 (8)	0.0046 (8)	−0.0006 (7)	−0.0020 (6)	0.0000 (6)
O5	0.0053 (9)	0.0244 (9)	0.0054 (8)	−0.0026 (8)	0.0008 (7)	0.0006 (6)

**Table 3:** Atomic distances (Å) in NaCu_2_VP_2_O_10_

Na–O1	2.861 (2)	(×2)		V–O2	1.649 (2)	(×2)
Na–O1	2.434 (2)	(×2)		V–O3	2.147 (2)	(×2)
Na–O2	2.670 (2)	(×2)		V–O4	1.973 (2)	(×2)
Na–O4	2.388 (2)	(×2)		〈V–O〉	1.9230	
〈Na–O〉	2.5883					
				P–O1	1.522 (2)	
Cu–O1	1.904 (2)			P–O3	1.516 (3)	
Cu–O2	2.060 (2)			P–O4	1.538 (2)	
Cu–O5	1.949 (2)			P–O5	1.561 (2)	
Cu–O5	1.972 (2)			〈P–O〉	1.5343	
〈Cu–O〉	1.9713					
