# Supplementary material for: Crystal structure and magnetism in the S = 1/2 spin dimer compound NaCu2VP2O10
Source: IUCrJ. 2020 May 27;7(Pt 4):656–62. doi: 10.1107/S2052252520005655 (PMC7340267; doi:10.1107/S2052252520005655)
Supplement: Supplementary file 3 [file m-07-00656-sup3.pdf]

IUCrJ

Volume 7 (2020)

Supporting information for article:

Crystal structure and magnetism in the *S* = 1/2 spin dimer compound $NaCu_2VP_2O_{10}$

Daisuke Urushihara, Sota Kawaguchi, Koichiro Fukuda and Toru Asaka

S1. Powder X-ray diffraction

Figures S1(a) and (b) show the powder XRD pattern and simulated diffraction pattern of $NaCu_2VP_2O_{10}$, respectively. All reflections are in good agreement with each pattern. Impurity phases were not observed at the resolution of the powder XRD measurement. We measured the magnetic susceptibility and heat capacity of this single-phase polycrystalline $NaCu_2VP_2O_{10}$ sample.

S2. Reciprocal sections using single-crystal X-ray diffraction

Figures S2(a), (b), and (c) show precession images obtained using single-crystal XRD of $NaCu_2VP_2O_{10}$. In Figs. S2(a) and (b), the 00l reflections follow the extinction rule of $l = 2n$ under kinematical diffraction conditions. In the SAED patterns (Fig. 1), the reflections with $l = 2n + 1$ in 00l were assumed to be multiple reflections because of the dynamical diffraction effects shown in Figs. S2(a) S2(b). Reciprocal sections determined using XRD were in good agreement with the SAED patterns considering multiple reflections. Both the XRD and SAED patterns suggested that the space group of $NaCu_2VP_2O_{10}$ was $C222_1$.

S3. Magnetic susceptibility fitting using a spin dimer model

Figure S3 displays the temperature dependence of the magnetic susceptibility of $NaCu_2VP_2O_{10}$. The isolated dimer model, which has only one exchange parameter J, does not match the measured susceptibility. In contrast, the alternating chain model is in good agreement with it. These results suggest that interdimer interactions are needed to describe the magnetic susceptibility of $NaCu_2VP_2O_{10}$.

S4. Heat capacity

Figure S4 displays the temperature dependence of the heat capacity of $NaCu_2VP_2O_{10}$. We did not observe any anomalies at low temperatures. This result suggests that $NaCu_2VP_2O_{10}$ shows a non-magnetic state without magnetic phase transitions.

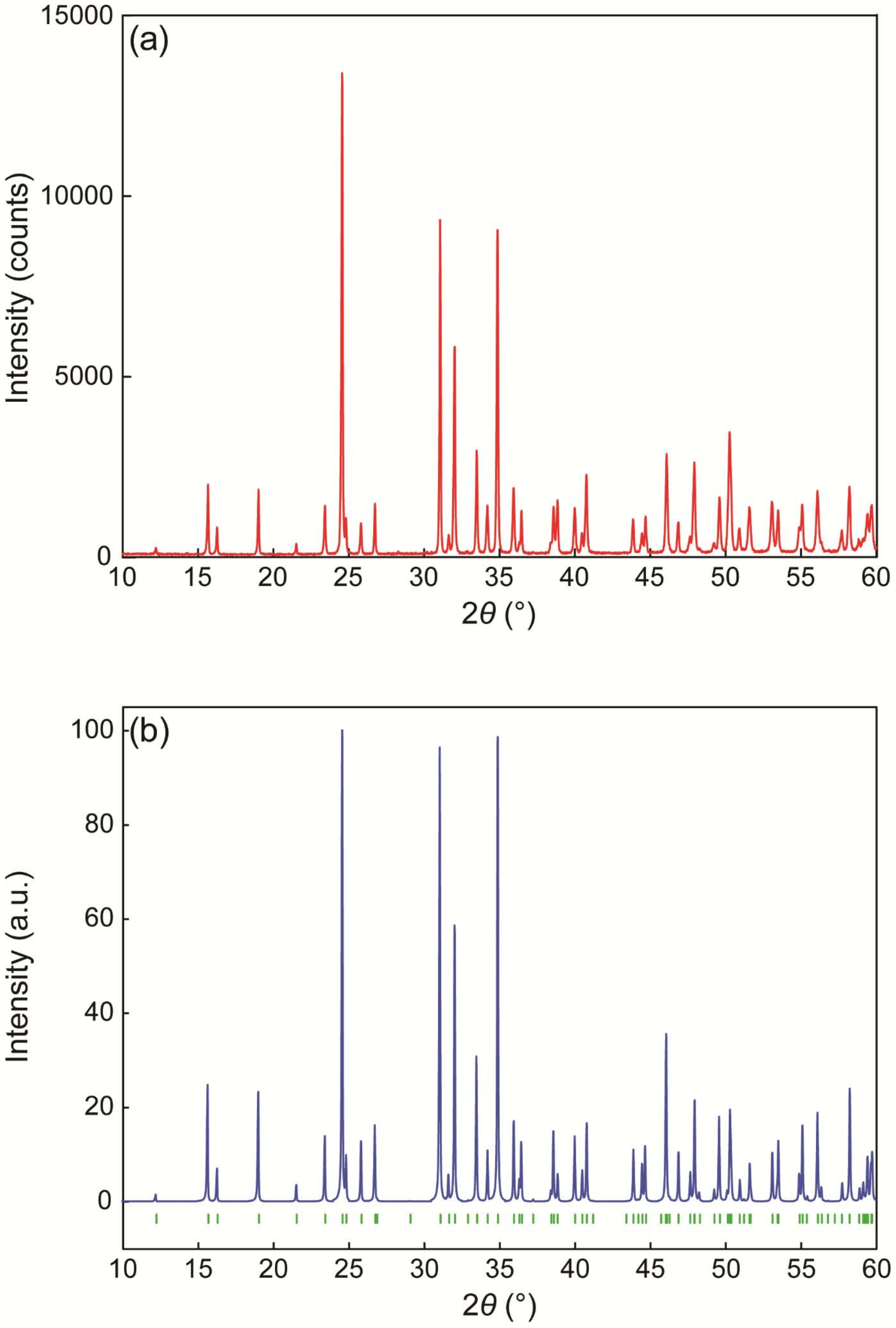

Figure S1 (a) Powder X-ray diffraction pattern and (b) simulated pattern of $NaCu_2VP_2O_{10}$.

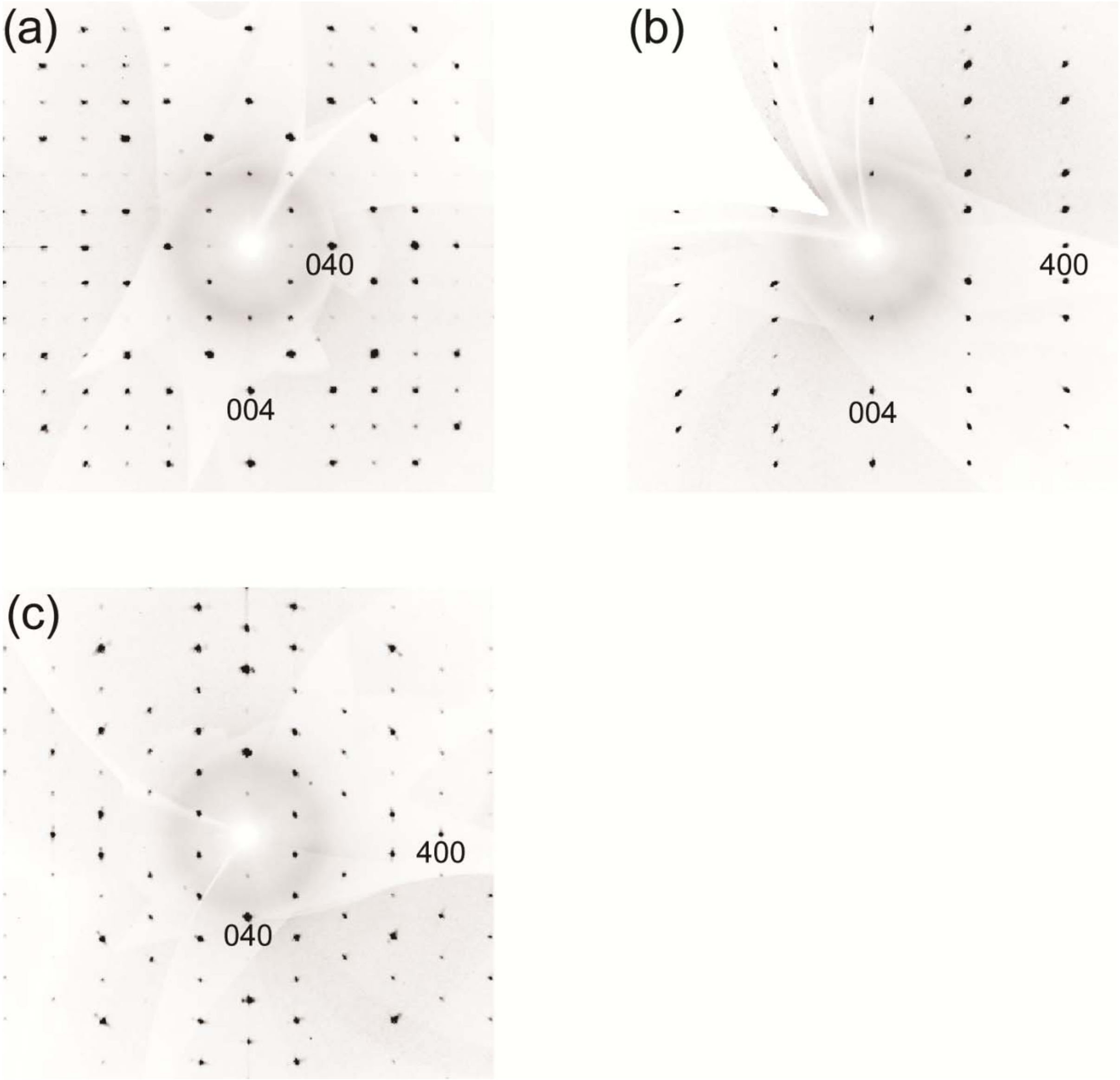

Figure S2 (a) 0*kl*, (b) *h*0*l,* and (c) *hk*0 reciprocal sections of $NaCu_2VP_2O_{10}$.

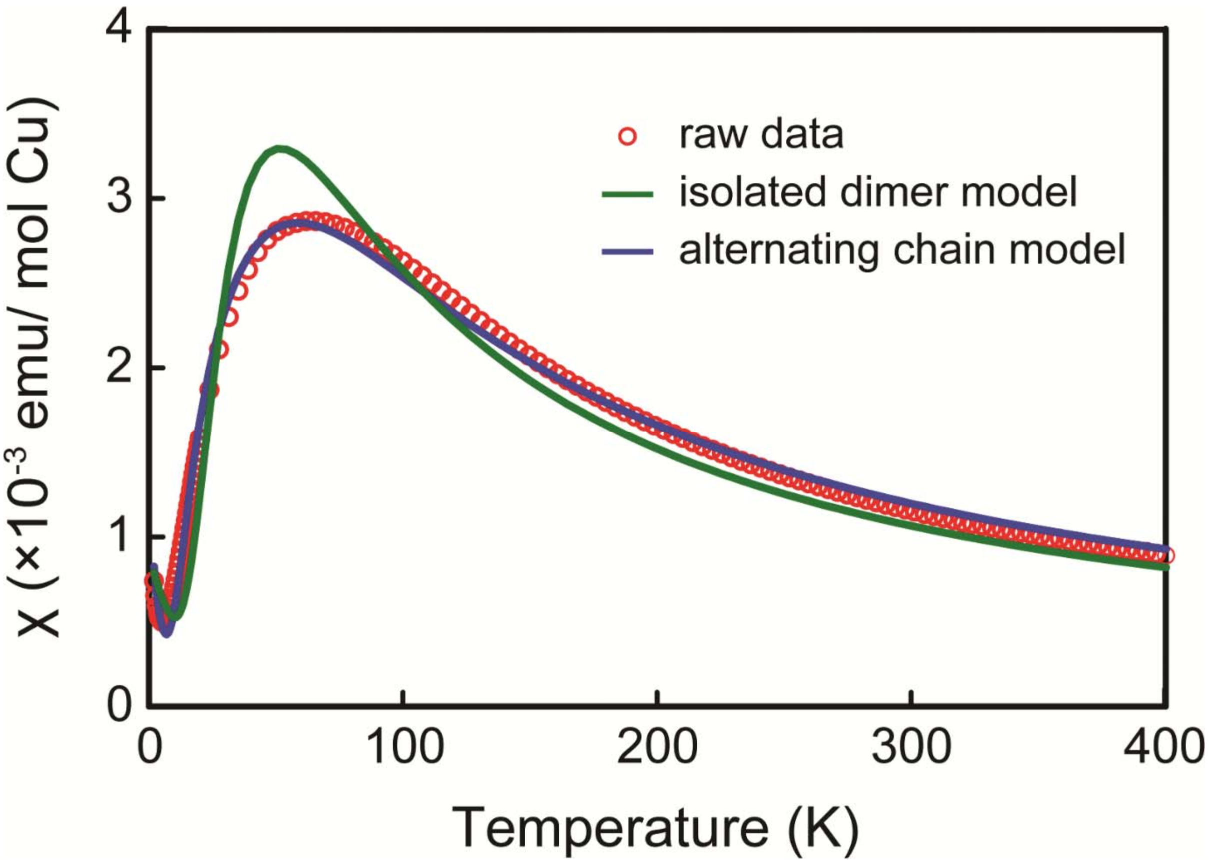

Figure S3 Temperature dependence of the magnetic susceptibility of $NaCu_2VP_2O_{10}$. The red open symbols represent raw data. The green and blue solid lines show the fitting curve of the isolated dimer and alternating chain models.

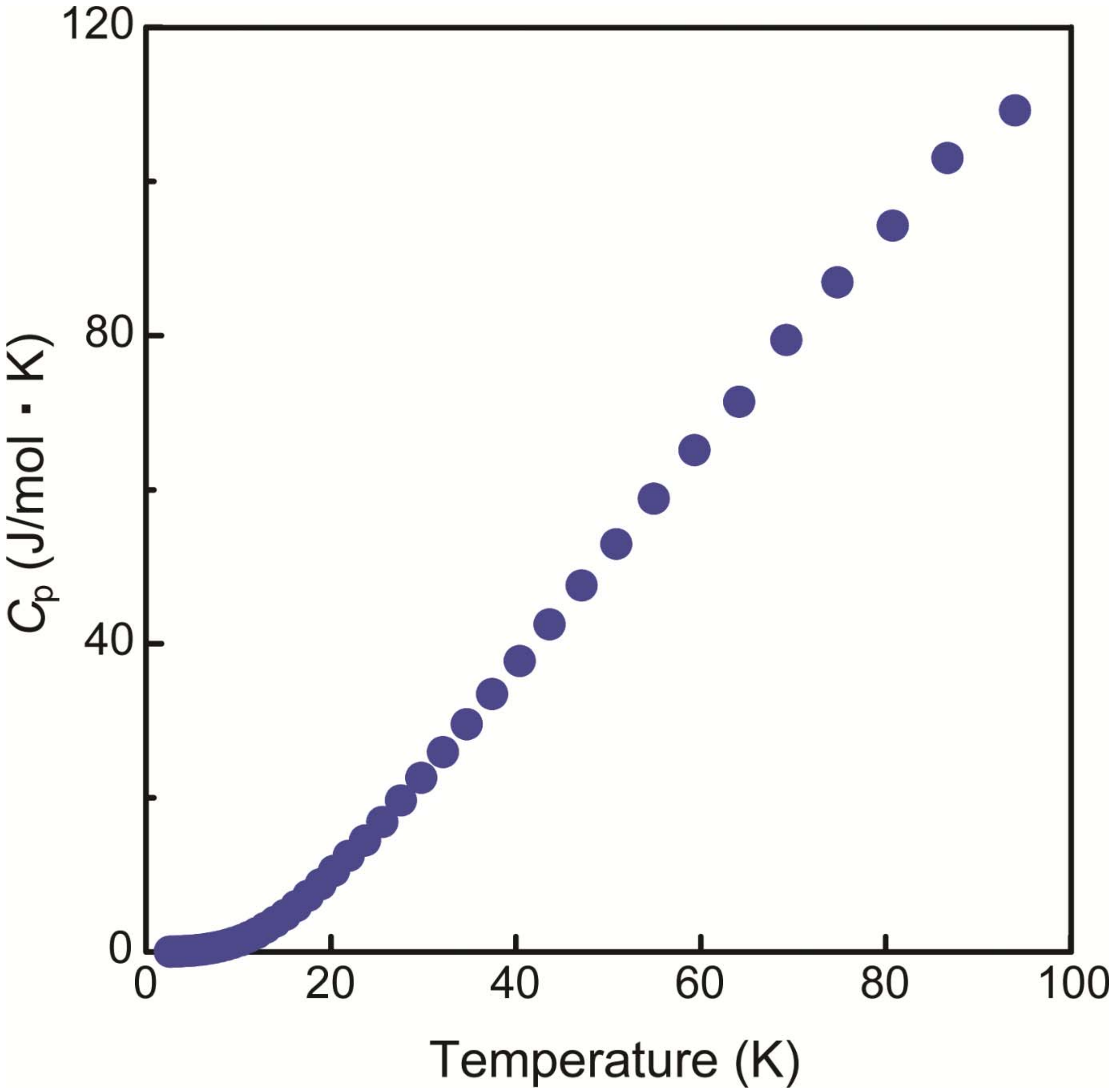

Figure S4 Temperature dependence of the heat capacity of $NaCu_2VP_2O_{10}$.
